# Machine Learning Prediction Models for Mitral Valve Repairability and Mitral Regurgitation Recurrence in Patients Undergoing Surgical Mitral Valve Repair

**DOI:** 10.3390/bioengineering8090117

**Published:** 2021-08-25

**Authors:** Marco Penso, Mauro Pepi, Valentina Mantegazza, Claudia Cefalù, Manuela Muratori, Laura Fusini, Paola Gripari, Sarah Ghulam Ali, Enrico G. Caiani, Gloria Tamborini

**Affiliations:** 1 Department of Cardiovascular Imaging, Centro Cardiologico Monzino IRCCS, 20138 Milan, Italy; Mauro.Pepi@cardiologicomonzino.it (M.P.); Valentina.Mantegazza@cardiologicomonzino.it (V.M.); Claudia.Cefalu@cardiologicomonzino.it (C.C.); Manuela.Muratori@cardiologicomonzino.it (M.M.); Laura.Fusini@cardiologicomonzino.it (L.F.); Paola.Gripari@cardiologicomonzino.it (P.G.); Sarah.Ghulamali@cardiologicomonzino.it (S.G.A.); Gloria.Tamborini@cardiologicomonzino.it (G.T.); 2 Department of Electronics, Information and Biomedical Engineering, Politecnico di Milano, 20133 Milan, Italy; enrico.caiani@polimi.it; 3 Consiglio Nazionale Delle Ricerche, Istituto di Elettronica e di Ingegneria dell’Informazione e Delle Telecomunicazioni, 20133 Milan, Italy

**Keywords:** mitral valve prolapse, machine learning, mitral valve repair, primary mitral regurgitation, prediction model, heart

## Abstract

Background: Mitral valve regurgitation (MR) is the most common valvular heart disease and current variables associated with MR recurrence are still controversial. We aim to develop a machine learning-based prognostic model to predict causes of mitral valve (MV) repair failure and MR recurrence. Methods: 1000 patients who underwent MV repair at our institution between 2008 and 2018 were enrolled. Patients were followed longitudinally for up to three years. Clinical and echocardiographic data were included in the analysis. Endpoints were MV repair surgical failure with consequent MV replacement or moderate/severe MR (>2+) recurrence at one-month and moderate/severe MR recurrence after three years. Results: 817 patients (DS1) had an echocardiographic examination at one-month while 295 (DS2) also had one at three years. Data were randomly divided into training (DS1: n = 654; DS2: n = 206) and validation (DS1: n = 164; DS2 n = 89) cohorts. For intra-operative or early MV repair failure assessment, the best area under the curve (AUC) was 0.75 and the complexity of mitral valve prolapse was the main predictor. In predicting moderate/severe recurrent MR at three years, the best AUC was 0.92 and residual MR at six months was the most important predictor. Conclusions: Machine learning algorithms may improve prognosis after MV repair procedure, thus improving indications for correct candidate selection for MV surgical repair.

## 1. Introduction

Mitral valve regurgitation (MR) is the most common valvular heart disorder [1] as part of the degenerative changes in the aging process and is associated with increased mortality [2]. Mitral valve prolapse (MVP) is a valvular heart disease disorder affecting about 2–3% of the general population and is frequently associated with severe MR requiring valve surgery [3,4]. This disorder is defined as an elongation or rupture of the chordal apparatus, resulting in leaflet prolapsing in the atrial cavity during ventricular contraction [5]. Although mitral valve (MV) repair represents the ideal procedure for MVP, with recognized advantages over MV replacement [6,7], patient outcome depends on multiple factors, such as pre-operative clinical and echocardiographic data, as well as surgical experience.

In this regard, recent guidelines state that in asymptomatic patients MV repair can be considered when there is a high likelihood of durable result with very low risk (<1% mortality) [8].

MV repair includes a combination of chordoplasty, leaflet resection, sliding valvuloplasty, commissuroplasty, Alfieri stitch repair, and/or annuloplasty with a complete or a partial ring [9]. Surgical outcomes depend on pre-operative status, technique of repair, and center experience. Although results have confirmed the high immediate operative success of mitral valvuloplasty (>95%) [10,11,12], degenerative MV repair can be expected after repair with consequent reoperation. The incidence of reoperation after initial MV surgical repair is 4.5–7% at five years, with a linearized hazard rate of 0.5–1.5% per year [11,13,14]. Especially elderly patients, due to more fragile tissues and poor preoperative ventricular function compared with younger patients, may not tolerate a failed repair [6]. The ability of predicting surgical or post-surgical MV repair failure may translate into a better selection of candidates and may result in better information to patients about their short- and long-term prognosis. There are many potential predictors for repairability and recurrence of MR relevant to clinical and surgical factors such as age, preoperative ventricular function, and surgical procedure complexity [11,12,13,15,16,17,18]. However, echocardiographic and surgical variables associated with MR recurrence are still controversial, suggesting that the underlying causes for MR recurrence need to be better understood [14,19]. Accordingly, to improve the outcome of initial MV repair it is important to analyze the causes and mechanisms of MV repair failure, also with new approaches. Recently, machine learning (ML) algorithms, as a subfield of artificial intelligence, were introduced to deal with the huge variability in clinical data, have shown promising results in different medical fields [20,21]. ML are excellent analytical methods for classification through identification of data patterns from complex data. Several ML models have been shown to accurately identify patients at high risk of mortality [22,23] with superiority as compared to statistical approaches for both classification and prediction in general medical settings [24,25,26]. However, prior works have not explored the potential of these methods to explain the causes of MV repair failure. We hypothesized that these approaches could be applied in order to identify new potential predictors, concurrently with a regression analysis, to allow a better comprehensive prediction of MV repair failure and MR recurrence.

In this work, we sought to develop a ML-based prognostic model, in synergy with a statistical regression model, for MV repair failure considering three different time windows: during surgery, at one-month (1M), and at three years (3Y) follow-up (FU). Accordingly, our contributions can be summarized as follows:

(1) Developing a feature selection technique using features importance ranking of non-linear methods to detect the most relevant predictors from input data.
(2) Evaluating different resampling techniques in order to balance the class distribution, which reflects the real world distribution of MVP.
(3) Performing an experimental analysis testing many well-known ML classification algorithms.
(4) Assessing an additive feature attribution method to improve interpretability of the ML outcomes and to provide a better understanding of data.

## 2. Materials and Methods

### 2.1. Study Population

Between 2008 and 2018, 1000 patients (age ≥ 18 years) with severe MR due to MVP underwent early MV surgery for MV repair at Centro Cardiologico Monzino IRCCS (Milan, Italy). Inclusion criteria for retrospective selection were a pre-operatory 2- and 3-D transthoracic echocardiography (TTE), as well as a 1M-FU 2DTTE. As a result, a dataset (DS1) of 817 patients was considered, with 13 patients excluded for poor 3DTTE quality, and 170 because 1M-FU 2DTTE was not available.

Within the surgical intervention, MV repair was considered successful by intraoperative TEE (Group 1: MR ≤ 2). If suboptimal (Group 2: MR > 2+), all patients underwent a second repair attempt or MV replacement.

Additionally, to investigate long term FU, a subgroup of 295 patients (DS2) was extracted from DS1 considering a successful MV repair and availability of results of 2DTTE examinations at six months (6M) and at 3Y-FU. Based on the results of MR at 3Y-FU, DS2 patients were further assigned to Group 3 (residual MR ≤ 2) or Group 4 (residual MR > 2).

The following clinical endpoints were considered: for DS1, MV repair surgical failure with consequent MV replacement, or moderate/severe MR (>2+) recurrence at 1M-FU; for DS2, moderate/severe MR recurrence after 3Y-FU.

The study was approved by the local ethical committee (R1168/20-CCM 1230), and all patients provided written informed consent.

### 2.2. Echocardiographic Measures

2DTTE and 3DTTE were performed using two ultrasound platforms (iE33 or EPIQ 7C). From 2DTTE, left ventricular (LV) end-diastolic and end-systolic volumes indexed for body surface area (BSA), LV ejection fraction, LV stroke volume (SV), left atrial area, and volume indexed for BSA (LAVI) were derived using the biplane Simpson’s method. Grading of tricuspid regurgitation (mild = 1, moderate = 2, severe = 3) was obtained according to guidelines [27]. Systolic pulmonary arterial pressure (PAPS) was calculated using the Doppler echocardiographic method [28]. The Carpentier nomenclature was applied to the MV leaflets [28]. MVP was defined as simple or complex: simple anatomical lesions included isolated P2 prolapse or P2 associated with P1 or P3. According to the literature [29,30], cases including lesions involving >2 posterior leaflet scallops, anterior or both leaflets, commissures or with severe annular or leaflet calcifications were defined as complex. The main phenotypes of MVP were also distinguished: Barlow disease (mixomatous leaflet degeneration, elongated and thickened chordae, dilated annulus) and fibroelastic deficiency (normal/thinner leaflets, frequent single segment prolapse with chordal rupture) [16,29]. To assess reproducibility of MVP evaluation (prolapsing scallop identification), intra-observer variability was performed by the same reader after ≥1 month, while inter-observer variability was performed by a second experienced reader blinded to the previous results. To determine the MVP gold standard interpretation, in case of discrepant evaluation between the two observers, consensus was reached also including a third observer in the evaluation. Echocardiographic MVP evaluation was compared with MV anatomical inspection performed by the operating surgeon. Protocols and reports of surgical techniques were annotated in detail. According to literature data [31,32] and surgical institutional experience, surgical procedures were divided into simple vs. complex techniques. A score of 1 (mild), 2 (mild-to-moderate), 3 (moderate-to-severe), or 4 (severe) was assigned to MR integrating both qualitative and quantitative parameters [27].

### 2.3. Study Design

**Figure 1:**
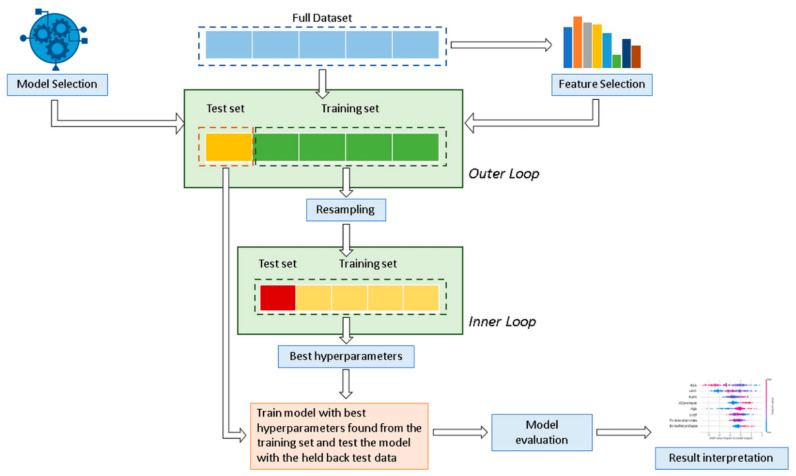
Overview of the proposed classification method for the mitral valve repair failure. A nested cross-validation is used to train a model in which hyperparameters also need to be optimized.

In this study, six well-known and regularly used supervised ML classification algorithms were applied and compared: decision tree (DT), random forest (RF), support vector machine (SVM), naive Bayes (NB), eXtreme gradient boosted trees (XGboost), and multilayer perceptron (MLP). In addition, data were also analyzed using a statistical logistic regression (LR) model. Figure 1 shows the analysis workflow schematically.

Data were divided into a training cohort (80% for DS1 and 70% for DS2), used to develop and train the ML algorithms and LR model, and a validation cohort (20% and 30%, respectively for DS1 and DS2) to ensure generalization of the model and prevent overfitting. Considering the relatively limited number of minority class patients available in the testing set, a stratified 20-fold Monte Carlo cross-validation (MCCV) [33] (Outer loop) was used for robust performance evaluation and to estimate model generalization. Cross validation is a common procedure to split data into a training set and a test set. MCCV represents an effective way to reduce the subsampling bias in the holdout procedure and the estimation variability in the test set [34,35]. The LR model was derived using a stepwise feature selection with backward elimination in a multivariate analysis for the identification of independent variables. Hyperparameters were determined by using a grid search analysis with stratified five-fold cross-validation on the training cohort (Inner loop) to optimize each ML model and determine the best value that led to the best performance in terms of *F_1_* score:

(1) $$F_{1}\;score=2\frac{\left(\frac{TP}{TP+FP}*\frac{TP}{TP+FN}\right)}{\left(\frac{TP}{TP+FP}+\frac{TP}{TP+FN}\right)}$$

where *TP* is the number of true positives and *FP* and *FN* are the number of false positives and false negatives, respectively. Further details on the model’s hyperparameters are presented in Table S1 in the Supplementary Materials. Model performance was assessed according to a range of learning metrics (positive and negative predictive values (PPV, NPV), mean area under the receiver operating characteristic curve (AUC)).

All analysis was performed by custom software using Python (Python Software Foundation, version 3.7) and scikit-learn package.

### 2.4. Pre-Processing

Nominal/ordinal variables were one-hot encoded, thus producing one binary feature for each category. To deal with the class imbalance distribution issue, several methods have been proposed in the literature, among which data level solutions are the most used techniques. An imbalance class creates a bias where the ML model tends to benefit the most frequent classes. The purpose of these techniques is to re-balance the class distribution by resampling the data to minimize the effect of the class imbalances on the training set. The resampling methods can be divided into two categories: oversampling and undersampling. Both are adopted to adjust the ratio between the different classes in the dataset. An oversampling approach, in order to balance the classes’ distribution, duplicated or synthesized some data from the minority class, while in an undersampling method some data of the majority class are removed. Table 1 shows a summary of classic binary resampling algorithms, reporting the strategy used, i.e., oversampling, undersampling or a combination of the two approaches (hybrid), and a description of the method. In order to balance the class distribution in the two datasets, all the reported methods in Table 1 were tested.

To deal with the potentially high number of noisy, redundant, and irrelevant variables that can slow down learning algorithms, leading to difficulties in interpretability of the models and even degenerating the performance of the learning task, different feature selection strategies were used, including dropping 0-variance features and highly correlated variables. ML involved automated feature selection by Gini importance ranking [42] as an indicator of feature relevance. Feature selection was performed using two ensemble tree-based ML algorithms, i.e., RF and XGboost and selecting the top ten features based on the rank of mean Gini importance over 100 iterations on all samples. The resulting selected features were subsequently used in the ML training phase, with recursive feature elimination for the identification of the most relevant features.

### 2.5. Clinical Assessment and Statistical Analysis

For model interpretability, evaluating how the computed features tend to influence the model prediction, an additive feature attribution method (Shapley Additive Explanations) was adopted [43], which defines a weighted linear regression by using data and predictions of the analyzed model. In addition, for decision-tree based models, to elucidate the level of importance for a specific feature, the average Gini index [42] was calculated. Continuous variables are presented as mean ± standard deviation, whereas categorical variables as absolute frequencies (%). Continuous variables were compared using the unpaired Student’s t-test, while categorical variables with the χ^2^ test or Fisher’s exact test, as appropriate. Models’ results were compared using Wilcoxon signed rank test or the DeLong test. Mean values in TTE parameters at baseline and at 6M-FU in each group of patients were compared with paired Student’s t-test. Inter- and intra-observer correlations were performed using Pearson’s coefficient. Statistical analyses were conducted with IBM SPSS 26 (SPSS Inc, Chicago, IL, USA), with *p*-values < 0.05 being considered significant.

## 3. Results

### 3.1. Cohort Characteristics

Table 2 describes the main clinical characteristics and TTE findings (baseline and/or 6M-FU) for DS1 and DS2. MV repair was performed in 767 pts (95.1%), while only 40 (4.9%) underwent MV replacement. Out of these 767 patients, 20 had MR>2 at 1M-FU.

Accordingly, the DS1 population was divided into 2 groups: Group 1 (757 cases: MR ≤ 2 at 1M-FU) and Group 2 (60 cases: MV replacement or MR > 2+ at 1M-FU). Patients in Group 2 were more likely to be older, with lower BSA and LV SV, higher PAPS, more complex MVP, more MV calcifications, and less MV chordae rupture, larger tricuspid annulus and tricuspid regurgitation. Inter- (r = 0.88, *p* < 0.001) and intra-observer (r = 0.96 *p* < 0.001) variability in echocardiographic MVP assessment resulted in a good agreement. The training (80%) and validation (20%) cohorts consisted of 654 patients (group 1: 605 and group 2: 49) and 164 patients (group 1: 152 and group 2: 12), respectively.

For the DS2, 268 patients (group 3, 90.8%) had no or mild MR (MR ≤ 2), while 27 (group 4, 9.2%) had MR > 2+ at 3Y-FU. Additionally, group 4 had higher PAPS (baseline and 6M-FU), LAVI (6M-FU), MR ≥ 2 (6M-FU), and more frequently underwent complex MV procedures. DS2 was split into 70% (group 3: 187 and group 4: 19) for the training cohort and 30% (group 3: 81, group 4: 8) for the validation cohort.

### 3.2. Selected Features

For DS1 the top predictors for MV repair failure and MR recurrence at 1M-FU shared across models were BSA, age, tricuspid valve diameter index, LAVI, and PAPS. For DS2, MR ≥ 2 (6M-FU), PAPS, and left atrial area had high importance in all models (further details in Table S2 in Supplement). From multivariate LR analysis (see Table 3), only the following predictors remained in the final model: for DS1, age, BSA, A2 prolapse and left atrial area; for DS2, P2 prolapse, PAPS, and MR ≥ 2 at 6M-FU.

### 3.3. Model Performance

Due to the large number of experimental results (>200 for both DS1 and DS2), we have decided to present only the best result for DS1 and DS2 (Table 4).

Among the six ML algorithms studied, the XGboost classifier presented the best results in all the prediction schemas, as well as for feature selection. These results were achieved using DS1 as the SVMSMOTE resampling algorithm and DS2 as the BorderlineSMOTE 1 algorithm. For DS1, after resampling, the initial distribution of the training set represented by 654 patients (group 1: 605 and group 2: 49) was transformed to 1210 patients (group 1: 605 and group 2: 605), whereas for DS2, the final training cohort was 374 patients (group 3: 187 and group 4: 187). Codes used for XGboost development are made publicly available in the Supplementary Materials.

**Figure 2:**
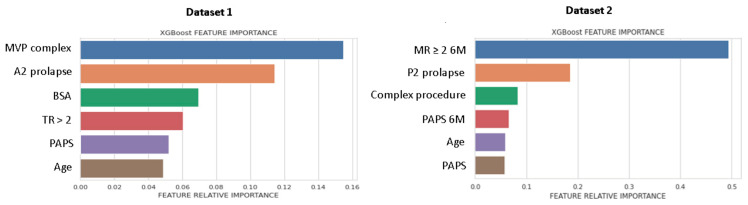
Feature importance plot for DS1 and DS2. Feature importance was measured as the Gini index. MVP, mitral valve prolapse; BSA, body surface area; TR, tricuspid regurgitation > 2; PAPS, systolic pulmonary artery pressure; MR, mitral valve regurgitation; 6M, six months.

Figure 2 shows the list of predictors included in the best performing ML-model, ranked for Gini importance. For DS1, morphological parameters had the greatest importance, while for DS2, the MR ≥ 2 6M-FU was the highest ranked predictor.

**Figure 3:**
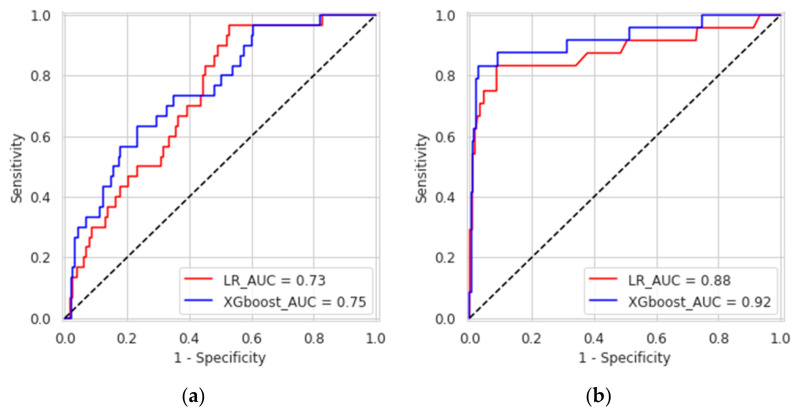
Receiver operating characteristic curves of the eXtreme gradient boosted trees and logistic regression for predicting causes of mitral valve repair failure and mitral valve regurgitation at one-month (**a**) and three years (**b**).

**Figure 4:**
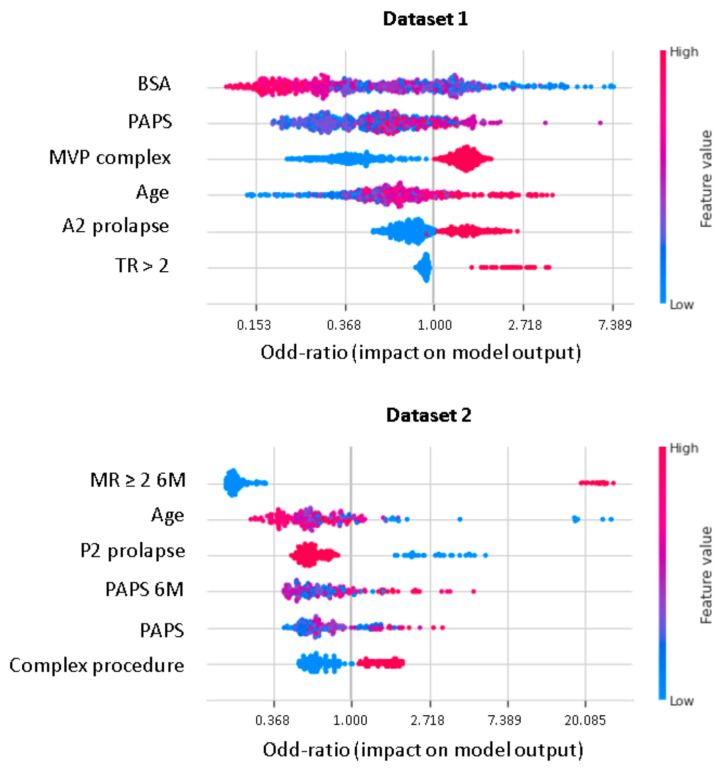
Shapley additive explanations value plot for DS1 and DS2. The horizontal axis shows whether the effect of the feature is associated with a higher or lower prediction, while the color indicates whether the value of the feature is high (red) or low (blue) for a given observation. MVP, mitral valve prolapse; BSA, body surface area; TR, tricuspid regurgitation > 2; PAPS, systolic pulmonary artery pressure; MR, mitral valve regurgitation; 6M, six months.

XGboost model obtained a better discrimination for MV repair failure than the LR, both in DS1 (AUC: XGboost 0.75 vs. LR 0.73) and DS2 (AUC: XGboost 0.92 vs. LR 0.88), as shown in Figure 3. For early MV repair failure identification, there was not a clear predominance among models in terms of PPV (PPV: XGboost 0.29 vs. LR 0.29). As regards the long-term follow-up for MR recurrence prediction, the best PPV was reached with the XGboost model (PPV: XGboost: 0.77 vs. LR: 0.70, *p* < 0.05). All the models had NPV of 0.95 or higher, and no improvement was observed in LR after resampling. RF and NB classifiers also consistently performed well, while other models were less robust to generalization. In Figure 4, for a global understating of the ML model, the effect of each feature on the learning-model prediction in terms of odd-ratio is shown.

## 4. Discussion

In this study, we developed and tested a ML-based prognostic model to predict the risk of MV repair failure and MR recurrence based on pre-operative clinical and echocardiographic data. We found that the XGboost presented the best discriminative abilities for the prediction of MV repair failure and/or MR recurrence at 1M-FU (AUC: 0.75) and at 3Y-FU (AUC: 0.92).

These findings suggest that in the future ML could have an important clinical role in evaluating prognostic risk in MVP patients undergoing MV repair. The ability to correctly identify MV repair failure and the time course of significant recurrent MR on the basis of preoperative clinical and echocardiographic parameters is challenging. Our results support the possibility of expanding indications and selection of cases undergoing MV repair.

XGboost and LR had similar discrimination on both datasets, emphasizing the possibility of combining the two approaches in order to further improve the prediction of outcomes. Although both ML and LR models had good PPV (which is potentially more clinically relevant than AUC) in predicting 3Y moderate/severe recurrent MR, they are less effective for an intra-operative or early MV repair failure assessment. Indeed, with DS1 the use of clinical TTE and MV morphology parameters was associated with a substantially lower PPV and AUC than with DS2.

ML classifiers, in contrast to regression-based classifiers, account for unexpected predictor variables and associations between features and outcomes, facilitating recognition of predictors not yet described in the literature [44]. However, ML models are more data dependent than conventional statistical techniques, requiring a larger sample size for a modelling technique to generate a classifier with a good discriminatory ability. Therefore, ML models need far more events per variable to achieve stable validated AUC than traditional LR models, which may be useful in relatively small datasets. Since in medicine, large, multidimensional, and balanced datasets are difficult to collect, resampling techniques are likely to become an essential tool for learning-applications in clinical practice, helping to train a better classifier in terms of performance and generalization.

Our study represents, to our knowledge, the first investigation comparing ML classifiers, including a regression-based model, to predict short- and long-term MV repair failure in a large MVP population. An accurate assessment of MV morphology was obtained by integrating the 3DTTE with the standard 2DTTE, which has been previously demonstrated to be feasible and accurate in the evaluation of MV anatomy in MVP patients [12]. The 3D transthoracic technique allows us to discriminate between simple and complex lesions and it showed superior accuracy in the identification of MV pathology compared to 2DTTE [45]. The majority of patients undergoing MV repair had a successful procedure (95,1%) in accordance with guidelines that suggest early repair only in an experienced center with high volume procedures, low mortality (<1%), and a repair rate of >95%.

As regards durability, MR recurrence increases long-term mortality and significantly worsens quality of life [46]. Guidelines define clinical and echocardiographic criteria for MV surgery in severe MR, but also state that in asymptomatic patients MV repair can be considered when there is a high likelihood of durable MV repair at low risk [47]. Javadikasgari et al. [30] demonstrated that MV repair was associated with less durability in complex disease. Repair of an anterior leaflet is more challenging than posterior leaflet, and it was associated with reduced durability [47]. Tamborini et al. [12] found that MVP anatomy and PAPS were predictors of residual MR. Advanced myxomatous changes with prolapse of both leaflets was recognized to influence failure of valve repair [48,49]. Moreover, recurrent MR after MV repair is associated with adverse LV remodeling [13]. A recent study showed that repair failure may occur in the aggressive resection of the posterior mitral leaflet [50]. Chordal shortening, implantation of artificial chordae, and no use of ring annuloplasty partially explain the recurrence of MR [51]. Our data showed that patients with complex degenerative MR, specifically with A2 prolapse, more frequently had MV replacement or MV repair early failure. Multivariate analysis also confirmed A2 prolapse as a potential predictor of intra- or post-operatory MV repair failure. Moreover, cases with more complex MVP and suboptimal results refer generally to elderly patients and had other pre-operative characteristics, such as a higher grade of tricuspid regurgitation and higher PAPS. In addition, according to regression analysis, left atrial size is a predictor of suboptimal outcome after surgery.

For heart chamber volumes, all cases without significant MR had favorable remodeling of LAVI and LV volume at 6M-FU, while in patients with residual MR > 2+ at 3Y-FU heart chamber volumes were not reduced and showed less favorable hemodynamics.

**Figure 5:**
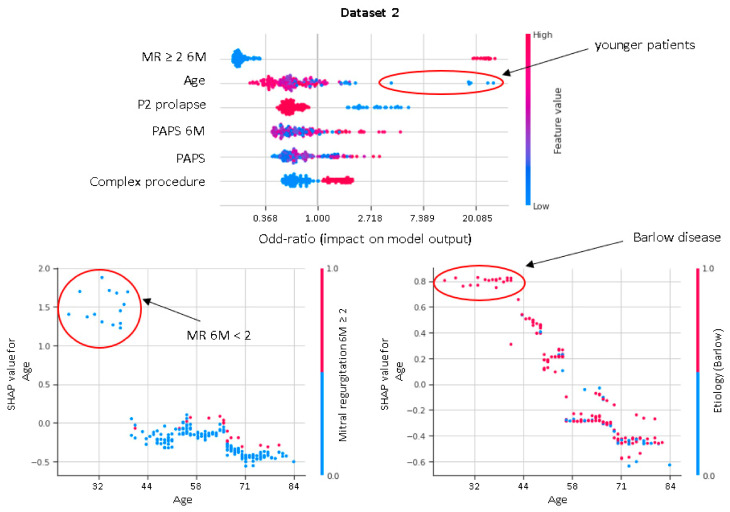
Shapley dependence plots. The horizontal axis shows the age. The color indicates whether the value of the second feature, which may have an interaction effect with the age, is high (red) or low (blue) for a given observation.

In our study, residual MR severity at 6M-FU represents the most important predictor of the durability at 3Y-FU of successful MV reconstruction. An increasing PAPS, along with a complex surgical procedure, is associated with a higher risk of suboptimal results at 3Y-FU. Interestingly, in younger patients there is a high likelihood of MR recurrence or MV replacement after MV repair. This is in accordance with previous studies and may be related to Barlow’s disease being associated with more complex MV apparatus anomalies affecting disease long term evolution (Figure 5).

Accurate identification of patients at high risk of short- and long-term MV repair failure is important for correct surgical planning. Our findings demonstrated that a ML approach based on an XGboost algorithm can predict MV repair failure at 3Y with good discrimination and significant higher PPV than LR, whereas predicting early surgical outcomes of MV repair procedures is more challenging and further research is needed. Indeed, while MVP is the most common valvular pathology requiring surgery, little is known about the genetic mechanism responsible for the pathogenesis and progression of the valvular disease. Previous publications have identified statistically significant predictors of repairability and recurrence of MR but, to our knowledge, without testing the model’s performance [11,12,13,15,16,17,18]. We have extended earlier studies in this domain by comparing a variety of models and variable pools. ML algorithms, in combination with the traditional regression approach, may offer a valid tool for expanding clinical knowledge and improving indications for correct candidate selection for MV surgical repair. These improvements may be furthered by the continuous gathering of patient data and the relatively easily way to retrain the model to account for new predictors. Consequently, it is expected that ML techniques will become increasingly important in the future to improve prognostics approaches. The method developed in this study can be extended even to other clinical challenges.

There are some limitations to the current study. First of all, the feasibility and performance of the ML approach were validated in a specific single-center population, which limits generalizability; the developed model will require prospective validation on a multicenter dataset. In addition, only variables that already existed in the dataset were considered in the modelling.

## 5. Conclusions

This study demonstrated that ML methods were able to predict MV repair success in MVP patients (intra-operative or early MV repair failure with AUC 0.75 and three years moderate/severe recurrent MR with AUC 0.92), thus representing an attractive tool, holding promises for integration into clinical workflow and forming a solid basis for further technical investigation and clinical studies.

## Figures and Tables

**Table 1:** Summary of classic binary resampling algorithms.

Resampling Algorithms		
Name	Strategy	Briefly Description
SMOTE [36]	Oversampling	Creates synthetic elements for the minority class, based on those that already exist, using a k-nearest neighbour algorithm.
SVMSMOTE [37]	Oversampling	Variant of SMOTE algorithm which uses an SVM algorithm to detect sample to use for generating new synthetic samples
BorderlineSMOTE 1 and 2 [38]	Oversampling	Creates synthetic samples from the minority class along the decision boundary between the two classes.
ADASYN [39]	Oversampling	Creates synthetic elements according to the data density.
ENN [40]	Undersampling	Remove samples close to the decision boundary using the edited nearest neighbour algorithm.
TomekLinks [40]	Undersampling	Removes overlap between classes.
SMOTEENN [41]	Hybrid	Combines SMOTE and ENN algorithms.
SMOTETomek [41]	Hybrid	Apply SMOTE and TomekLinks algorithms.

**Table 2:** Clinical, procedural, and echocardiographic characteristics of the study population.

Characteristics	Dataset 1			Dataset 2		
	Group 1	Group 2	*p*-Value	Group 3	Group 4	*p*-Value
	(n = 757)	(n = 60)		(n = 268)	(n = 27)	
Age, years	61.1 ± 12.2	67.7 ± 11.6	<0.001	59.8 ± 12.5	63.3 ± 15.3	0.183
Female, n(%)	89(11.8%)	12(20%)	0.062	88(32.8%)	10(37.0%)	0.668
Body surface area, m^2^	1.84 ± 0.19	1.72 ± 0.18	<0.001	1.82 ± 0.19	1.81 ± 0.22	0.805
MVP etiology			0.206			0.154
FED	219(28.9%)	22(36.7%)		68(25.4%)	3(11.1%)	
Barlow	538(71.1%)	38(63.3%)		200(74.6%)	24(88.9%)	
Atrial fibrillation	89(11.8%)	12(20.0%)	0.062	21(7.8%)	3(11.1%)	0.472
Complex MV prolapse	392(51.8%)	45(75%)	0.001	130(48.5%)	17(63.0%)	0.157
Complex surgical procedure	286(37.8%)	25(41.7%)	0.620	83(31.0%)	16(59.3%)	0.003
Antero-posterior mitral annulus diameter (mm)	36.65 ± 5.20	36.10 ± 5.20	0.429	37.05 ± 5.26	36.15 ± 5.09	0.394
Medio-lateral mitral annulus diameter (mm)	39.78 ± 5.00	38.83 ± 4.98	0.155	39.77 ± 4.91	38.56 ± 4.36	0.217
Prolapse						
P1	84(11.1%)	6(10.0%)	0.794	20(7.5%)	3(11.1%)	0.455
P2	652(86.1%)	41(68.3%)	<0.001	237(88.4%)	18(66.7%)	0.001
P3	174(23.0%)	18(30.0%)	0.217	60(22.4%)	6(22.2%)	0.976
A1	44(5.8%)	10(16.7%)	0.001	16(6.0%)	2(7.4%)	0.675
A2	204(26.9%)	29(48.3%)	<0.001	74(27.6%)	9(33.3%)	0.537
A3	154(20.3%)	16(26.7%)	0.315	52(19.4%)	6(22.2%)	0.733
Bi-leaflet prolapse	178(23.5%)	20(33.3%)	0.088	67(25.0%)	6(22.2%)	0.742
Commissure						
Anterolateral	13(1.7%)	1(1.7%)	1.000	2(0.7%)	1(3.7%)	0.252
Posteromedial	105(13.9%)	12(20.0%)	0.192	34(12.7%)	5(18.5%)	0.377
Calcification	152(20.1%)	19(31.7%)	0.034	52(19.4%)	7(25.9%)	0.425
Cleft	75(9.9%)	6(10.0%)	0.982	27(10.1%)	4(14.8%)	0.506
Ruptured chordae	567(74.9%)	34(56.7%)	0.002	201(75.0%)	24(88.9%)	0.152
LVEDV index (mL/m^2^)	77.2 ± 18.9	73.0 ± 16.5	0.090	77.0 ± 19.1	80.7 ± 20.9	0.342
LVESV index (mL/m^2^)	27.5 ± 9.1	26.6 ± 7.5	0.477	26.8 ± 9.4	29.1 ± 10.7	0.239
LVSV index (mL/m^2^)	49.8 ± 12.7	46.4 ± 11.8	0.048	50.2 ± 12.8	51.6 ± 12.9	0.582
LVEF (%)	64.5 ± 6.7	63.4 ± 6.4	0.219	65.3 ± 6.8	64.6 ± 7.1	0.606
LA area (cm^2^)	29.6 ± 7.4	30.3 ± 7.8	0.510	29.9 ± 7.4	32.3 ± 7.6	0.115
LA volume index (mL/m^2^)	57.6 ± 22.1	59.4 ± 22.1	0.540	64.3 ± 24.4	66.7 ± 27.2	0.628
PAPS (mmHg)	36 ± 10	40 ± 13	0.002	35 ± 10	46 ± 18	0.006
Tricuspid valve diameter index (mm/m^2^)	19.5 ± 2.7	20.6 ± 3.4	0.004	19.6 ± 2.8	20.9 ± 4.9	0.187
Tricuspid regurgitation > 2	40(5.3%)	9(15.0%)	0.002	18(6.7%)	4(14.8%)	0.130
Mitral regurgitation > 3	709(93.7%)	51(85.0%)	0.011	249(92.9%)	24(88.9%)	0.424
Tricuspid valvuloplasty	103(13.6%)	11(18.3%)	0.309	44(16.4%)	8(29.6%)	0.088
Aortic valve			0.653			0.531
Repair	16(2.1%)	2(3.3%)		6(2.3%)	0(0%)	
Replacement	6(0.8%)	0(0%)		6(2.3%)	0(0%)	
Ascending aorta	6(0.8%)	0(0%)	1.000	3(1.1%)	0(0%)	1.000
CABG	45(5.9%)	1(1.7%)	0.244	28(10.4%)	3(11.1%)	1.000
PFO closure	7(0.9%)	1(1.7%)	0.458	6(2.3%)	1(3.7%)	0.494
Left atrial appendage closure	20(2.6%)	1(1.7%)	1.000	16(6.0%)	1(3.7%)	1.000
Atrial fibrillation ablation	27(3.6%)	0(0%)	0.253	21(7.8%)	2(7.4%)	1.000
LVEDV index 6M (mL/m^2^)				57.0 ± 14.8 *	61.6 ± 16.5 *	0.124
LVESV index 6M (mL/m^2^)				24.4 ± 10.7 *	26.4 ± 10.6 *	0.360
LVSV index 6M (mL/m^2^)				32.5 ± 7.6 *	35.2 ± 9.4 *	0.087
LVEF 6M (%)				58.0 ± 7.9 *	57.9 ± 9.6	0.953
LA area 6M (cm^2^)				22.6 ± 5.6 *	25.9 ± 5.3 *	0.003
LA volume index 6M (mL/m^2^)				44.3 ± 17.5 *	53.4 ± 18.5 *	0.011
PAPS 6M (mmHg)				28 ± 6 *	31 ± 8	0.025
Mitral regurgitation 6M ≥ 2				10(3.7%)	19(70.4%)	<0.001

Values are mean ± SD or n (%). CABG, coronary artery bypass graft; EDV, end diastolic volume; EF, ejection fraction; ESV, end systolic volume; FED, fibroelastic deficiency; LA, left atrial; LV, left ventricular; MV, mitral valve; PAPS, systolic pulmonary artery pressure; PFO, patent foramen ovale; SV, stroke volume; 6M, six months. *: *p* < 0.05, Baseline vs. six months (paired *t*-test).

**Table 3:** Multivariate logistic regression analysis.

**Dataset 1**		
	Multivariate	
	OR (95% CI)	*p*-value
Age, years	1.051 (1.023–1.080)	<0.001
Body surface area	0.080 (0.018–0.361)	0.001
A2 prolapse	2.757 (1.582–4.805)	<0.001
Left atrial area	1.033 (1.021–1.067)	0.047
**Dataset 2**		
	Multivariate	
	OR (95% CI)	*p*-value
P2 prolapse	0.190 (0.055–0.654)	<0.001
Systolic pulmonary artery pressure	1.053 (1.010–1.099)	0.001
Mitral regurgitation 6M ≥2	53.761 (16.666–173.421)	<0.001

**Table 4:** Classifiers’ predictive performance in the validation cohort for the two datasets.

**Dataset 1**					
Algorithm	PPV	NPV	AUC	Resampling	Feature selection
DT	0.16	0.95	0.61	BorderlineSMOTE 1	Random Forest
RF	0.24	0.95	0.69	BorderlineSMOTE 1	Random Forest
SVM	0.18	0.95	0.64	SVMSMOTE	eXtreme Gradient boosted
NB	0.16	0.96	0.71	SMOTEENN	eXtreme Gradient boosted
XGboost	0.29	0.96	0.75	SVMSMOTE	eXtreme Gradient boosted
MLP	0.19	0.96	0.65	BorderlineSMOTE 1	eXtreme Gradient boosted
LR	0.29	0.95	0.73	/	Multivariate logistic regression
**Dataset 2**					
Algorithm	PPV	NPV	AUC	Resampling	Feature selection
DT	0.41	0.96	0.78	BorderlineSMOTE 1	Random Forest
RF	0.64	0.97	0.88	BorderlineSMOTE 1	Random Forest
SVM	0.45	0.96	0.80	SVMSMOTE	eXtreme Gradient boosted
NB	0.65	0.97	0.89	SVMSMOTE	eXtreme Gradient boosted
XGboost	0.77	0.97	0.92	BorderlineSMOTE 1	eXtreme Gradient boosted
MLP	0.64	0.96	0.83	BorderlineSMOTE 1	eXtreme Gradient boosted
LR	0.70	0.97	0.88	/	Multivariate logistic regression

AUC, area under the receiver operating characteristic curve; DT, decision tree; LR, logistic regression; MLP, multilayer perceptron; NB, naive Bayes; NPV, negative predictive value; PPV, positive predictive value; RF, random forest; SVM, support vector machine; XGboost, eXtreme gradient boosted trees.

## Data Availability

The data presented in this study are not publicly available due to privacy and ethical restrictions.

## Supplementary Materials

The following are available online at https://www.mdpi.com/article/10.3390/bioengineering8090117/s1. Table S1: Hyperparameters for decision tree, random forest, support vector machine, gradient boosting and multilayer perceptron algorithms; Table S2: Feature selection. Top ten variables’ importance in descending order for random forest and gradient boosting models.
