# Supplementary material for: Machine Learning Prediction Models for Mitral Valve Repairability and Mitral Regurgitation Recurrence in Patients Undergoing Surgical Mitral Valve Repair
Source: Bioengineering (Basel). 2021 Aug 25;8(9):117. doi: 10.3390/bioengineering8090117 (PMC8469985; doi:10.3390/bioengineering8090117)
Supplement: Supplementary file 1 [file bioengineering-08-00117-s001.zip › bioengineering-1285109-supplementary.pdf]

Supplementary data of:

Machine Learning Prediction Models for Mitral Valve Repairability and Mitral Regurgitation Recurrence in Patients Undergoing Surgical Mitral Valve Repair

Marco Penso [1,2]*, Mauro Pepi [1], Valentina Mantegazza [1], Claudia Cefalù [1], Manuela Muratori [1], Laura Fusini [1,2],
Paola Gripari [1], Sarah Ghulam Ali [1], Enrico G. Caiani [2,3] and Gloria Tamborini [1]

1 Department of Cardiovascular Imaging, Centro Cardiologico Monzino IRCCS, 20138 Milan, Italy; Mauro.Pepi@cardiologicomonzino.it (M.P.); Valentina.Mantegazza@cardiologicomonzino.it (V.M.); Claudia.Cefalu@cardiologicomonzino.it (C.C.); Manuela.Muratori@cardiologicomonzino.it (M.M.); Laura.Fusini@cardiologicomonzino.it (L.F.); Paola.Gripari@cardiologicomonzino.it (P.G.); Sarah.Ghulamali@cardiologicomonzino.it (S.G.A.); Gloria.Tamborini@cardiologicomonzino.it (G.T.)
2 Department of Electronics, Information and Biomedical Engineering, Politecnico di Milano, 20133 Milan, Italy; enrico.caiani@polimi.it
3 Consiglio Nazionale Delle Ricerche, Istituto di Elettronica e di Ingegneria dell'Informazione e Delle Telecomunicazioni, 20133 Milan, Italy
* Correspondence: Marco.Penso@cardiologicomonzino.it; Tel.: +39-392-693-0900

Table S1 – Hyperparameters for decision tree, random forest, support vector machine, gradient boosting and multilayer perceptron algorithms.

Hyperparameter tuning of the models was conducted by grid search using five-fold cross-validation. The scoring function used was F1-score. The hyper-parameter space searched over are presented below.

**Decision Tree**	
criterion	Gini
max_depth	[3-8]
min_samples_split	[2-7]
min_samples_leaf	[1-4]
**Random Forest**	
criterion	Gini
n_estimators	[50,60,80,100,200,400,500]
max_depth	[3-8]
min_sample_split	[3-8]
min_samples_leaf	[1-4]
**Support Vector Machine**	
C	[0.1, 1,10,100]
kernel	['poly', 'rbf', 'sigmoid']
gamma	[1, 0.1, 0.01, 0.001,'auto']
probability	True
**Gradient boosting machine**	
learning_rate	[0.1,0.01]
n_estimators	[50,60,80,100,200,400,500]
max_depth	[3-8]
min_child_weight	[1-4]
gamma	[0, 0.5,1,1.5,2,5]
colsample_bytree	[0.6,0.8,1]
**Multilayer perceptron**	
batch size	[1,2,4,8]
hidden layer	[1-3]

learning rate	[0.1,0.01,0.001]
dropout rate	[0.0,0.2,0.3,0.5]
dense layer units	[5,10,15]
weight initialization	['uniform', 'zero', 'he_normal', 'he_uniform']
activation function	Rectified linear unit

Table S2 – Feature selection. Top ten variables importance in descending order for Random Forest and Gradient Boosting models. Gini importance ranking was used to evaluate the worth of each variable by measuring the total decrease in node impurity averaged over all trees of the ensemble model.

**Dataset 1**	
Random forest	eXtreme Gradient boosted
Age	A2 prolapse
Body surface area	Age
Tricuspid valve diameter index	Tricuspid regurgitation
Left atrial volume index	Body surface area
Left ventricular stroke volume index	Complex MV prolapse
Left ventricular ejection fraction	P2 prolapse
Systolic pulmonary artery pressure	Left atrial volume index
Left ventricular end systolic volume index	Systolic pulmonary artery pressure
Left atrial area	A1 prolapse
Medio-lateral mitral annulus diameter	Tricuspid valve diameter index
**Dataset 2**	
Random forest	eXtreme Gradient boosted
Mitral regurgitation 6M ≥2	Mitral regurgitation 6M ≥2
Systolic pulmonary artery pressure	Systolic pulmonary artery pressure
Age	P2 prolapse
Tricuspid valve diameter index	Posteromedial commissure
Left atrial area 6M	Age
Left atrial volume index 6M	Left atrial area 6M
Left ventricular ejection fraction 6M	Left atrial area
Left ventricular end systolic volume index	Systolic pulmonary artery pressure 6M
Systolic pulmonary artery pressure 6M	Complex surgical procedure
Left ventricular stroke volume index 6M	MV prolapse etiology (Barlow)

MV, mitral valve; 6M, six months.

```
# Removing Constant Features using Variance Threshold
print('initial columns: %d' % len(x.columns))
constant_filter = VarianceThreshold(threshold=0.01)
constant_filter.fit(x)
constant_columns = [column for column in x.columns
                    if column not in x.columns[constant_filter.get_support()
]]
print('0 variace columns: %d' % len(constant_columns))
for column in constant_columns:
    x = x.drop([column],1)
    print(column)
print(x.shape)



#Removing Correlated Features using corr() Method
dset = pd.concat([x, y1], axis=1, sort=False)
correlated_features = set()
correlation_matrix = dset.corr()
leng = len(correlation_matrix)
for i in range(len(correlation_matrix .columns)):
    for j in range(i):
        if abs(correlation_matrix.iloc[i, j]) > 0.7:
            print(correlation_matrix.columns[i],'-
', correlation_matrix.columns[j], '-', abs(correlation_matrix.iloc[i, j]))
            if abs(correlation_matrix.iloc[i, leng-
1]) > abs(correlation_matrix.iloc[j, leng-1]):
                colname = correlation_matrix.columns[j]
                correlated_features.add(colname)
            elif abs(correlation_matrix.iloc[j, leng-
1]) > abs(correlation_matrix.iloc[i, leng-1]):
                  colname = correlation_matrix.columns[i]
                  correlated_features.add(colname)
            elif abs(correlation_matrix.iloc[i, leng-
1]) == abs(correlation_matrix.iloc[j, leng-1]):
                if correlation_matrix.iloc[i, leng-
1] > correlation_matrix.iloc[j, leng-1]:
                    colname = correlation_matrix.columns[j]
                    correlated_features.add(colname)
                else:
                    colname = correlation_matrix.columns[i]
                    correlated_features.add(colname)
print(correlated_features)

#feature selection method

# apply threshold to positive probabilities to create labels
def to_labels(pos_probs, threshold):
  return (pos_probs >= threshold).astype('int')
```

```
def adjusted_classes(y_scores, th):
    """
    This function adjusts class predictions based on the prediction threshol
d.
    Will only work for binary classification problems.
    """
    return [1 if y >= th else 0 for y in y_scores]

features = x.columns
scaler = MinMaxScaler()
label_encoder = LabelEncoder()
n_iterations = 100
feature_importance_values_RF = np.zeros(len(features))
feature_importance_values_GB = np.zeros(len(features))

for i in range(n_iterations):
    print('iteration number: %d' % i)
    dtX = dt.drop(['out1'],1)
    dtY = dt['out1']
    dtX = dtX.values
    stY = dtY.values
    dtX = scaler.fit_transform(dtX)
    dtY = label_encoder.fit_transform(dtY)

    modelRF = RandomForestClassifier(n_estimators=800, max_depth=7)
    #modelLGB = lgb.LGBMClassifier(objective ='binary', n_estimators=1000, l
earning_rate = 0.05, max_depth=5, min_child_samples=2)
    modelXGB = xgb.XGBClassifier(objective="binary:logistic", n_estimators=8
00, learning_rate=0.01, max_depth=7, colsample_bytree=1)

    fittedRF = modelRF.fit(dtX, dtY)
    fittedGB = modelXGB.fit(dtX, dtY)

    # Record the features importance
    feature_importance_values_RF += fittedRF.feature_importances_ / n_iterat
ions
    feature_importance_values_GB += fittedGB.feature_importances_ / n_iterat
ions


feature_importances = pd.DataFrame({'feature': features, 'importance': featu
re_importance_values_RF})
# Sort features according to importance
feature_importances = feature_importances.sort_values('importance', ascendin
g = False).reset_index(drop = True)
# Normalize the feature importances
feature_importances['normalized_importance'] = feature_importances['importan
ce'] / feature_importances['importance'].sum()
# Extract the features with zero importance
```

```
record_zero_importance = feature_importances[feature_importances['importance
'] == 0.0]
to_drop = list(record_zero_importance['feature'])
# plot features impotances
plot_n = 10
plt.figure(figsize = (10, 8))
ax = plt.subplot()
ax.barh(list(reversed(list(feature_importances.index[:plot_n]))),
        feature_importances['normalized_importance'][:plot_n],
        align = 'center', edgecolor = 'k')
ax.set_yticks(list(reversed(list(feature_importances.index[:plot_n]))))
ax.set_yticklabels(feature_importances['feature'][:plot_n], size = 12)
# Plot labeling
plt.xlabel('Normalized Importance', size = 16); plt.title('Feature Importanc
es RF', size = 18)
plt.show()
```

```
feature_importances = pd.DataFrame({'feature': features, 'importance': featu
re_importance_values_GB})
# Sort features according to importance
feature_importances = feature_importances.sort_values('importance', ascendin
g = False).reset_index(drop = True)
# Normalize the feature importances
feature_importances['normalized_importance'] = feature_importances['importan
ce'] / feature_importances['importance'].sum()
# Extract the features with zero importance
record_zero_importance = feature_importances[feature_importances['importance
'] == 0.0]
to_drop = list(record_zero_importance['feature'])
# plot features impotances
plot_n = 10
plt.figure(figsize = (10, 8))
ax = plt.subplot()
ax.barh(list(reversed(list(feature_importances.index[:plot_n]))),
        feature_importances['normalized_importance'][:plot_n],
        align = 'center', edgecolor = 'k')
ax.set_yticks(list(reversed(list(feature_importances.index[:plot_n]))))
ax.set_yticklabels(feature_importances['feature'][:plot_n], size = 12)
# Plot labeling
plt.xlabel('Normalized Importance', size = 16); plt.title('Feature Importanc
es GB', size = 18)
plt.show()
```

```
# apply threshold to positive probabilities to create labels
def to_labels(pos_probs, threshold):
    return (pos_probs >= threshold).astype('int')
```

```
def adjusted_classes(y_scores, t):
    """
    This function adjusts class predictions based on the prediction threshol
d (t).
    Will only work for binary classification problems.
    """
    return [1 if y >= t else 0 for y in y_scores]

for i in range(n_iterations):
    print ('------------------------------------')
    print(i)
    train, test = train_test_split(dset, test_size=0.30, random_state=i, str
atify=dset['out1'])


model = xgb.XGBClassifier(objective="binary:logistic)
    param_grid = {'min_child_weight': [1, 2, 3, 4],
                  'gamma': [0.5, 0, 1, 1.5, 2, 5],
                  'colsample_bytree': [0.6, 0.8, 1],
                  'max_depth': [3, 4, 5, 6, 7, 8],
                  'learning_rate': [0.1, 0.01],
                  'n_estimators': [50,60,80,100,200,400,500]

                  }
    skf = StratifiedKFold(n_splits=5,shuffle=True, random_state=0)
    skf.get_n_splits(x_train, y_train)
    gs = GridSearchCV(
        estimator=model,
        param_grid=param_grid,
        cv=skf,
        n_jobs=-1,
        scoring='f1',     #roc_auc    recall    f1
        refit=True
    )
    fitted_model1 = gs.fit(x_train, y_train)
    print('Best parameters: {}'.format(gs.best_params_))
    print('Best score: {}'.format(gs.best_score_))
    fitted_model1 = fitted_model1.best_estimator_
    pred_prob1 = fitted_model1.predict_proba(x_test)
    pred1 = fitted_model1.predict(x_test)
    XGBytrue.append(y_test)
    XGBypred_prob.append(pred_prob1[:,1])
    XGBypred.append(pred1)
    # define thresholds
    thresholds = np.arange(0, 1, 0.001)
    # evaluate each threshold
    scores = [f1_score(y_test, to_labels(pred_prob1[:,1], th)) for th in thr
esholds]
    # get best threshold
    ix = np.argmax(scores)
```

```
    XGBthres.append(thresholds[ix])
    pred_adj = adjusted_classes(pred_prob1[:,1], thresholds[ix])
    # precision
    precision = precision_score(y_test, pred_adj)
    print('Precision: %.2f' % precision)
    XGBcvprecision.append(precision)
    # recall
    recall = recall_score(y_test, pred_adj)
    print('Recall: %.2f' % recall)
    XGBcvrecall.append(recall)
    # f1
    f1 = f1_score(y_test, pred_adj)
    print('f1: %.2f' % f1)
    XGBcvf1.append(f1)
    # accuracy
    acc = accuracy_score(y_test, pred_adj)
    print('acc: %.2f' % acc)
    XGBcvacc.append(acc)
    # ROC AUC
    auc = roc_auc_score(y_test, pred_prob1[:,1])
    print('ROC AUC: %f' % auc)
    XGBcvauc.append(auc)
    # confusion matrix
    matrix = confusion_matrix(y_test, pred_adj)
    print(matrix)
    TN = matrix[0,0]
    FP = matrix[0,1]
    FN = matrix[1,0]
    TP = matrix[1,1]
    XGB_TP.append(TP)
    XGB_TN.append(TN)
    XGB_FP.append(FP)
    XGB_FN.append(FN)
    print('FP %d' % FP)
    print('FN %d' % FN)
    print('TP %d' % TP)
    print('TN %d' % TN)
    XGB_PPV.append(TP/(TP+FP) )
    XGB_NPV.append(TN/(FN+TN) )
    XGB_specificity.append(TN/ (FP+TN) )
    feature_importance_values_XGB += fitted_model1.feature_importances_ / n_
iterations

#plot
feature_importance = feature_importance_values_XGB
feature_names = np.array(features)
#Create a DataFrame using a Dictionary
data={'feature_names':feature_names,'feature_importance':feature_importance}
fi_df = pd.DataFrame(data)
#Sort the DataFrame in order decreasing feature importance
```

```python
fi_df.sort_values(by=['feature_importance'], ascending=False,inplace=True)
#Define size of bar plot
plt.figure(figsize=(8,5))
#Plot Searborn bar chart
sns.barplot(x=fi_df['feature_importance'], y=fi_df['feature_names'])
#Add chart labels
plt.title('XGBoost ' + 'FEATURE IMPORTANCE')
plt.xlabel('FEATURE RELATIVE IMPORTANCE')
plt.ylabel('FEATURE NAMES')
```
